# p90 ribosomal S6 kinase: a potential therapeutic target in lung cancer

**DOI:** 10.1186/s12967-016-0768-1

**Published:** 2016-01-14

**Authors:** Noufira Poomakkoth, Aya Issa, Nabeel Abdulrahman, Somaia Gamal Abdelaziz, Fatima Mraiche

**Affiliations:** College of Pharmacy, Qatar University, P.O. Box 2713, Doha, Qatar

**Keywords:** p90 ribosomal S6 kinase, Lung adenocarcinomas, Mitogen activated protein kinases, Epidermal growth factor

## Abstract

A global survey of cancer has shown that lung cancer is the most common cause of the new cancer cases and cancer deaths in men worldwide. The mortality from lung cancer is more than the combined mortality from breast, prostate and colorectal cancers. The two major histological types of lung cancer are non-small cell lung cancer (NSCLC) accounting for about 85 % of cases and small cell lung cancer accounting for 15 % of cases. NSCLC, the more prevalent form of lung cancer, is often diagnosed at an advanced stage and has a very poor prognosis. Many factors have been shown to contribute to the development of lung cancer in humans including tobacco smoking, exposure to environmental carcinogens (asbestos, or radon) and genetic factors. Despite the advances in treatment, lung cancer remains one of the leading causes of cancer death worldwide. Interestingly, the overall 5 year survival from lung cancer has not changed appreciably in the past 25 years. For this reason, novel and more effective treatments and strategies for NSCLC are critically needed. p90 ribosomal S6 kinase (RSK), a serine threonine kinase that lies downstream of the Ras–MAPK (mitogen activated protein kinase) cascade, has been demonstrated to be involved in the regulation of cell proliferation in various malignancies through indirect (e.g., modulation of transcription factors) or direct effects on the cell-cycle machinery. Increased expression of RSK has been demonstrated in various cancers, including lung cancer. This review focuses on the role of RSK in lung cancer and its potential therapeutic application.

## Background

Lung cancer has emerged as a major public health problem and is the leading cause of cancer related death in both men and women worldwide [1, 2]. The expected number of lung cancer deaths in the U.S in 2015 is 158,040 [1]. Unfortunately, standard treatment modalities such as chemotherapy, radiotherapy, and surgery have reached a plateau [3]. Therefore, research efforts to identify alternatives to the conventional treatment are needed. A better understanding of the molecular origin and pathophysiology of lung cancer are essential to developing novel molecular targets for the treatment and prevention of lung cancer.

Two major forms of lung carcinoma exist including non-small cell lung cancer (NSCLC), which consists of approximately 85 % of all lung cancers and small cell lung cancer (SCLC), which accounts for 15 % of all lung cancers. The 5 year survival rate of NSCLC is about 18 % [4] and for small cell lung cancer is 6 % [5]. The three major histologic subtypes of NSCLC include adenocarcinomas, squamous cell carcinoma and large cell lung cancer. Adenocarcinomas, the most common histological variant seen in non-smokers and the one which presents with the best prognosis, accounts for 40 % of all lung cancers [6]. It is also the most common variant seen in females and adults less than 60 years of age [7]. This review focuses on the molecular mechanism and potential therapeutic targets for lung cancer, with emphasis on lung adenocarcinomas.

## Review

### Molecular origin of lung cancer

The origin of lung cancer is a multistep process induced by genetic and epigenetic alterations. These genetic and epigenetic alterations result in DNA damage and cellular adaptations to tolerate the oncogenic changes [8]. The cellular adaptations attributed to lung cancer include self-sufficient growth signals due to the occurrence of mutations in proto-oncogenes, lack of sensitivity to anti-proliferating signals as a result of mutations in tumor suppressor genes, evasion of apoptosis, unlimited replicative potential, detachment of tumor cells from the extracellular matrix, which leads to invasion of surrounding tissue and basal lamina. Tumor cells also have the capacity of sustained angiogenesis and they transport through the blood stream and migrate to distant sites, leading to the formation of a metastatic lesion [8, 9]. Cancerous cells have also been demonstrated to have a reversed pH gradient compared with normal adult cells. They exhibit a constitutively higher intracellular pH (pH_i_) and a lower extracellular pH (pH_e_) [10]. This increased pH_i_ favors cell proliferation, cell survival, evasion from apoptosis, cell migration and promotes tumor invasion [10]. An understanding of the mutated oncogenes, genetic alterations and the cellular adaptations has paved the road for identifying molecular therapeutic targets.

#### Epidermal growth factor and the epidermal growth factor receptor in lung adenocarcinomas

Epidermal growth factor receptor (EGFR) is overexpressed in 32–81 % of NSCLC [11]. EGFR plays a major role in activating several downstream signaling pathways like Ras/Raf/MEK/MAPK and the pathway consisting of phosphoinositide 3-kinase (PI3K), Akt, and the mammalian target of rapamycin (mTOR). Activation of these major downstream signaling pathways contribute to the cell proliferation, increased survival, invasiveness, metastatic spread, and angiogenesis in tumor cells [2]. Numerous therapeutics agents are available to target EGFR in NSCLC including Erlotinib, Geftinib and Cetuximab. Despite the importance of EGFR in mediating NSCLC, many of the available therapeutic agents targeting EGFR are ineffective [11]. Acquired resistance to the anti-EGFR agents also results from secondary mutations specifically in the exon 20 of the EGFR gene [3, 8]. Recent studies have also suggested that Ras mutations in lung adenocarcinomas were found to be associated with resistance to EGFR tyrosine kinase inhibitors (TKI) [3]. In addition, persistent activity of the mitogen activated protein kinase/extracellular-signal-regulated kinase (MAPK/ERK) pathway and the PI3K/Akt kinase pathway could contribute to the resistance of NSCLC to EGFR inhibitors [12]. Other proposed resistance mechanisms to EGFR inhibitors include amplification of the MET proto-oncogene, which activates PI3K pathway independent of the EGFR [13] and activation of other tyrosine kinase receptors such as the insulin like growth factor receptor 1 [2]. This has directed research activity towards identifying other molecular targets like Ras, Raf, MAPK and ERK, which may be beneficial in the management of lung adenocarcinomas.

#### Ras proto-oncogene

The Ras proto-oncogene plays an important role in the transduction of growth promoting signals from the cell membrane to the nucleus and the resulting cell proliferation [9]. The Ras proto-oncogene family (KRas, HRas, NRas and RRas) encodes four highly homologous 21 kDa membrane-bound proteins. Proteins encoded by the Ras genes exist in two states: an active state, in which GTP is bound to the Ras and an inactive state, where the GTP has been cleaved to GDP through the intrinsic GTPase activity [3]. The GTPase Ras activates Raf (A-, B- and C-Raf isoforms) [14]. The signal for cell proliferation is ultimately transmitted by a cascade of RAS-dependent kinases, which activates the MAPKs. It is noteworthy that 15–30 % of lung adenocarcinomas harbor activating mutations in the Ras family members, especially the KRas [3]. Mutations in the Ras induces defects in the intrinsic GTPase activity of Ras resulting in continuous cell proliferation [9]. The importance of KRas in lung carcinomas makes it a promising therapeutic target [8, 15]. However, Ras inhibitors (farnesyl transferase inhibitors) which inhibit post-translational modification and membrane localization of Ras proteins have been unsuccessful in clinical trials. This could be attributed to the fact that these inhibitors are not selectively active in tumors with KRas or NRas mutations [8, 15]. Recent research has focused on investigating downstream effectors of RAS including MAPKs, since they control fewer of the downstream pathways [8, 16].

#### Mitogen activated protein kinases and lung adenocarcinomas

The MAPK/ERK pathway is activated by various extracellular stimuli including mitogens, cytokines, growth factors and cellular stresses [17]. The binding of EGF to the EGFR activates the Ras proto-oncogene, which then activates the Raf kinase. In turn, Raf phosphorylates and activates the MAPK/ERK kinase (MEK)1/2, a dual-specificity protein kinase, which activate ERK1/2. Once activated, the ERK1/2 phosphorylates several substrates including members of the RSK (90 kDa ribosomal S6 kinase) family [14]. The Ras–MAPK also activates the PI3K/AKT pathway that regulates the normal cell proliferation, survival, growth and differentiation [8].

Mutations or overexpression of many of the signaling components in the MAPK pathway can confer oncogenic potential and lead to several human cancers [17, 18]. Activation of the Ras/Raf/MEK/MAPK via activating mutations in KRas occurs in approximately 30 % of adenocarcinomas [3]. Activation of downstream signaling pathways such as PI3K and MAPK occur independent of the EGFR signaling, therefore, rendering KRas mutant tumors resistant to anti EGFR agents and chemotherapy [19]. Therefore, targeting the downstream effectors of this pathway represent an untapped pool of possible therapeutic targets in the treatment of lung cancer. Currently, two MEK1/2 inhibitors, selumetinib and trametinib, have been tested in many different cancer types including NSCLC [17]. Selumetinib, which is the agent furthest in development raised a concerning rate of hospitalization, grade 3 or 4 neutropenia, and febrile neutropenia [17]. Therefore, research efforts have been directed to identifying a small set of effectors such as RSK, which are less likely to cause severe adverse effects [16, 20].

### RSK family of kinases

The efforts to identify the kinase activity responsible for the phosphorylation of ribosomal protein S6 (rpS6), led to the purification of an intracellular kinase that phosphorylated 40S ribosomal subunit from unfertilized Xenopus laevis eggs by Erikson and Maller laboratories in 1985. This kinase was initially referred to as ribosomal S6 kinase (S6K) [21]. The identification of two protein kinases of 85–90 kDa (S6KI and S6KII) by biochemical purification, led to the cloning of cDNAs encoding highly homologous proteins that were later renamed p90 ribosomal S6 kinase [14]. The RSK family of proteins comprises a group of highly conserved serine/threonine kinases that lie downstream of the Ras–MAPK pathway and regulate diverse cellular processes such as cell growth and motility, cell proliferation and cell survival [14, 18].

#### Structure of RSK

The structure of RSK is characterized by two distinct kinase domains separated by a linker region of about 100 amino acids and flanked by N- and C-terminal ends [18]. The RSKs are 73–80 % identical with each other and are mostly divergent in their N- and C-terminal sequences [14, 22]. The carboxyl-terminal kinase domain (CTKD) is closely related to the calcium/calmodulin-dependent protein kinase (CAMK) family. In contrast, the amino-terminal kinase domain (NTKD) is homologous to that of AGC kinases. The CTKD is responsible for auto-phosphorylation of RSK and the NTKD is involved in substrate phosphorylation [18]. Finally, the C-terminal region contains an ERK1/2 docking site also known as the D-domain, which is responsible for the docking and activation of RSK by ERK1/2 [22].

#### RSK family

In humans, the RSK family comprises of four isoforms (RSK1 to −4) and two structurally related cousins, called RSK-like protein kinase/mitogen and stress activated kinase-1 (RLPK/MSK1) and RSK-B (MSK2) [22]. Analysis of the expression patterns of RSK isoforms showed that RSK1 mRNA are more abundant in the lung, kidney, pancreas, bone marrow and T cells. RSK2 mRNA are predominantly found in T cells, lymph nodes, and the prostate. RSK3 transcripts are mainly expressed in the lung, brain, spinal cord, and retina. Interestingly, RSK4 mRNA expression in both adult and embryonic tissues is much lower than that of the other three isoforms. But Northern blotting of lysates from adult mouse tissues has revealed the expression of RSK4 mRNA in the brain, cerebellum, heart, renal tissue and skeletal muscle [18, 22].

#### Activators of RSK

RSKs are directly phosphorylated and activated by ERK1/2 and phosphoinositide dependent protein kinase 1 (PDK1) in response to various stimuli including growth factors, neurotransmitters and phorbol esters [18]. The MSKs are potently activated by both the ERK1/2 and the p38 pathways and are generally thought to be more responsive to cellular stress [18, 23]. Unlike the RSKs, MSK is usually located in the nucleus of cells and phosphorylates transcription factors [24]. Mutational analysis revealed that four phosphorylation sites (Ser221, Ser363, Ser380, and Thr573 in human RSK1) are essential for RSK activation upon mitogenic stimulation [10, 19]. The phosphorylation of Thr573 in the CTKD occurs following ERK activation. This activation also requires ERK docking at the D domain. The Ser380 is auto-phosphorylated by the activated CTKD [18]. Phosphorylation of Ser221 in the NTKD is mediated by PDK1 for RSK1–3, which leads to complete activation of RSK. This is further emphasized in PDK1 deficient cells, where mitogens do not stimulate RSK1–3 activity [14]. Once activated the RSKs may remain associated with the membrane, or in the cytosol or translocate to the nucleus, and eventually can phosphorylate substrates throughout the cell [18].

#### Biological function of RSK isoforms

The biological function of the RSK isoforms is to regulate cell-cycle progression and cell proliferation, cell growth and protein synthesis, nuclear signaling, cell migration and cell survival [14, 23].

#### Activation of cytosolic and nuclear proteins through phosphorylated RSK

Activation of the RSK protein kinase results in the phosphorylation of functionally diverse RSK substrates in the cytosol and nucleus. In the cytosol, phosphorylated RSK substrates include glycogen synthase kinase 3 (GSK3), protein phosphatase 1, LK B1, L1 CAM (a neural cell adhesion molecule), the Ras exchange factor; and membrane-associated tyrosine and threonine specific cyclin dependent kinase 1 or cell division cycle protein [17]. Nuclear translocation of phosphorylated RSK following mitogenic stimulation leads to phosphorylation of a variety of transcription factors including CREB, CREB binding protein (CBP), serum response factor (SRF), p300, ER81, oestrogen receptor-α (ERα), c-Fos, nuclear factor-Κb (NF-κB), NFATc4, NFAT3 and the transcription initiation factor TIF1A [23, 24]. Activation of these cytosolic and nuclear proteins contributes to the initiation and progression of tumorgenesis [23].

#### RSK and cell cycle machinery

RSKs are involved in the regulation of cell-cycle progression through phosphorylation of several mediators of the cell-cycle machinery. RSK mediated phosphorylation inactivates membrane-associated tyrosine and threonine specific CDC2 inhibitory kinase-1 (Myt1), leading to G2–M cell-cycle progression [18]. RSK1 and RSK2 have also been shown to promote G1-phase progression by phosphorylating the cyclin-dependent kinase (CDK) inhibitor p27^KIP1^. In addition, RSK phosphorylates serum response factor (SRF) and contributes to the transcriptional activation of c-FOS. Activation of c-FOS results in the activation of cyclin D1, promoting G1–S phase progression [18]. RSK phosphorylates and inhibits glycogen synthase kinase (GSK3), which has been suggested to promote stabilization of cyclin D1 and MYC, resulting in cell cycle progression and cell survival [18, 23]. In addition, RSK phosphorylates eEF2K and the translation-initiation factor eIF4B, which in turn stimulates its recruitment to the translation-initiation complex and contributes to cell growth and survival [14]. The phosphorylation of transcription factors such as CREB by RSK1 and RSK2 promotes cell survival by activating pro-survival genes such as members of the B cell lymphoma protein-2 (Bcl2) family [22]. Clearly, activation of RSK is critical in the phosphorylation of numerous mediators involved in the cell-cycle machinery.

#### RSK and apoptosis

High levels of endogenous Bcl2 are expressed in several lung cancer cell lines including those from NSCLC and SCLC [9]. The fate of these cancer cells is largely dependent on the balance between inhibitory and stimulatory apoptosis signals from the Bcl2 family. The subfamily members including Bcl2, Bcl-XL, and Mcl-1 inhibit apoptosis, whereas the Bax subfamily, consisting of Bax and Bak, as well as the BH3-only subfamily, including Bad, Bid, Bok, Bik, and Bim, promotes apoptosis [25].

RSK enhances cell survival via anti-apoptotic mechanisms [16, 22]. RSK phosphorylates the pro-apoptotic protein Bad and enhances its binding to 14-3-3 proteins. This prevents Bad from antagonizing the pro-survival function of Bcl-XL [22]. The RSK mediated phosphorylation of the death associated protein kinase (DAPK) leads to inhibition of its pro-apoptotic function [26]. RSK1/2 mediated phosphorylation of a tumor suppressor gene Bim-EL prevents its pro-apoptotic function [22, 27]. RSK1 directly inhibits caspase activity leading to increased cell survival [28]. Taken together, these data indicate that RSKs are invariably involved in cell proliferation and survival, making them promising therapeutic targets for the treatment of cancer.

### RSK and lung cancer

RSKs have been demonstrated to be over expressed or hyper activated in several cancers including breast cancer, lung cancer, prostate cancer, head and neck squamous cell carcinoma, ovarian carcinoma, multiple myeloma, melanoma and osteosarcoma [16, 29]. Different RSK isoforms behave differently depending on the type of cancer.

Activation or overexpression of RSK in lung cancer cells inhibits cell death via inactivation of the pro-apoptotic protein Bad [16]. Similarly, Bim-EL, which is sequentially phosphorylated by ERK and RSK1 or RSK2, is decreased in NSCLC cells with EGFR-activating mutations. The decrease in Bim-EL results in proteosomal degradation of BIM-EL and increased cell survival [30]. When the H-Ras/ERK pathway is activated in tumor cells, BIM-EL is eliminated by proteasomal degradation [27]. Additionally the expression of DAPKm which behaves as a tumor suppressor, is commonly silenced in lung cancer through DNA methylation [14]. Lara and colleagues observed that RSK4 is overexpressed in more than 50 % of primary malignant lung cancers though its levels are undetectable in normal cells [22]. Interesting, a previous report by Lara et al. demonstrated that the knock down of p90RSK isoform 1 enhanced the metastatic potential of A549 lung adenocarcinoma cells. Similarly, an siRNA kinome library screen in A549 cells demonstrated that p90RSK isoform 1 silencing increased migration and invasion [30]. Moreover, Lara et al. [29] reported that there is an increased migration in A549 cells caused by RSK 2 and 4. Clearly, the exact role and signaling pathway of the respective RSK isoforms in lung cancer remain unknown.

### RSK inhibitors

The identification of RSK inhibitors has uncovered an unexpected link between RSK activity and cell proliferation. Several pan-RSK small-molecule inhibitors exist including two competitive inhibitors that target the NTKD (SL-0101 and BI-D1870) and an irreversible inhibitor of the CTKD, FMK [22]. The first specific inhibitor identified for p90 RSK was SL0101, which was isolated from the tropical plant *Forsteronia refracta*. When tested against a panel of 70 kinases, it was shown to target RSK1 and RSK2 in the nanomolar range (IC_50_ for RSK2, 90 nmol/L at 10 mmol/L ATP) while having no significant activity against other tested AGC kinases [28]. The dihydropteridinone BI-D1870 is a reversible inhibitor that competes with ATP by binding to the NTKD ATP-interacting sequence. BI-D1870 is remarkably selective for RSK relative to other AGC kinases [31] and its in vitro IC_50_ was shown to be approximately 15–30 nM at an ATP concentration of 100 *μ*M [32]. *In vivo* results indicate that to completely inhibit the phosphorylation of RSK substrates in vitro, a concentration of 10 *μ*M BI-D1870 is required [32]. The pyrrolopyrimidine FMK (fluoromethylketone) is an irreversible inhibitor that covalently modifies the CTKD of RSK1, RSK2 and RSK4. FMK is a potent and specific inhibitor of RSK and was shown to inhibit RSK2 at an IC_50_ of 15 nM and an EC_50_ of 200 nM in vitro [33]. Recently another CTKD inhibitor, dibenzyl trisulfide, has been isolated from the *Petiveria alliacea L* plant. It specifically inhibits the RSK1 isoform at a concentration of 10 µM [20]. Therefore, the discovery of RSK-specific inhibitors will definitely help to advance the knowledge of RSK-mediated mechanisms in lung cancer and to test the potential of these inhibitors in pre-clinical studies. Our own unpublished data suggests that exposure of A549 lung adenocarcinoma cells to BI-D1870 decreases RSK1 protein expression and is associated with a decrease in cell migration and proliferation. Indeed, with the discovery of RSK-specific inhibitors further studies will need to be carried to verify the efficacy of RSK inhibitors as single agents or in combination with other anti-cancer agents in the lung cancer setting.

## Conclusion

RSKs are an important downstream effector of the Ras–Raf–MAPK signaling pathway. They play a crucial role in the regulation of cellular proliferation, growth, and survival in a variety of tumors. Based on our recent advances in the understanding of the different isoforms of RSK and the mechanisms by which they affect tumorgenesis, invasion and metastasis these agents might prove to be promising targets in the chemotherapy of lung adenocarcinomas particularly those harboring oncogenic mutations in components of the Ras signaling pathway.
